# A Case of Pyomyoma following Uterine Fibroid Embolization and a Review of the Literature

**DOI:** 10.1155/2016/9835412

**Published:** 2016-03-15

**Authors:** Chika C. Obele, Samantha Dunham, Genevieve Bennett, Johanna Pagan, Lok Yun Sung, Hearns W. Charles

**Affiliations:** ^1^Department of Radiology, Tisch Hospital, NYU School of Medicine, 660 First Avenue, 3rd Floor, New York, NY 10016, USA; ^2^Department of Obstetrics and Gynecology, Tisch Hospital, NYU School of Medicine, 111 Broadway 2nd Floor, New York, NY 10006, USA; ^3^Division of Vascular & Interventional Radiology, Department of Radiology, Tisch Hospital, NYU School of Medicine, 660 First Avenue, 7th Floor, New York, NY 10016, USA; ^4^Department of Radiology, North Shore University Hospital, Hofstra University School of Medicine, 300 Community Drive, Manhasset, NY 11030, USA

## Abstract

*Background.* Since its introduction in 1996, uterine fibroid embolization (UFE) has become standard medical practice in the management of symptomatic uterine fibroids. An extremely rare complication, pyomyoma, has been reported only 5 times previously in the literature following UFE.* Case.* A 37-year-old woman underwent UFE for symptomatic leiomyomas of the uterus. Signs and symptoms of uterine infection ensued, beginning at 6 days following the procedure. Recurrent fevers and increasing leukocytosis despite the intravenous administration of appropriate antibiotics eventually necessitated surgical intervention on postprocedure day #18.* Conclusion.* An extremely rare complication of UFE is herein presented, pyomyoma, with a review of other reported cases. Commonalities are sought among these few reported cases with the hope of increasing diagnostic acumen in the detection of this disease.

## 1. Introduction

Uterine leiomyoma is the most common benign tumor of the uterus and a common indication for uterine artery embolization (UAE) for patients wishing to have an alternative to medical and surgical management. Severe complications from fibroid embolization are rare. One of the most rarely reported complications may be pyomyoma. A suppurative leiomyoma, pyomyoma, has been reported to occur spontaneously, and also in postpartum, postinstrumentation, and postsurgery states. There have been 67 cases reported in the literature since 1871, with 13.6% mortality rate after the introduction of antibiotic in 1945 [1]. Most reported cases are related to pregnancy or gynecological procedures. Between 1945 and 2010, only 22 cases of pyomyoma have been reported in the literature and confirmed on postoperative or postmortem pathology. Postembolization infections in general are often related to ischemia of the uterus or leiomyomas. Pyomyomas are caused by infarction, necrosis, and infection of a leiomyoma [2]. In this report, we present a case of a 37-year-old woman who developed pyomyoma after uterine fibroid embolization (UFE).

## 2. Case Report

Institutional review board approval is not required for this type of case reporting at our institution.

A 37-year-old G0P0 woman with chronic severe anemia had persistent severe menometrorrhagia, requiring her to change her tampon and pads every 15 minutes during the menstrual cycle. She also had symptoms of urinary frequency, presumably secondary to her enlarged myomatous uterus. The patient had a myomectomy 8 years prior to presentation and had received Lupron for approximately 1.5 years, without clinical success. There was no history of pelvic inflammatory disease and there was no concomitant diabetes mellitus. The patient was seen in Vascular and Interventional Radiology (VIR) clinic and, due to her young age and childless state, she was advised to undergo a second myomectomy, but she refused. Due to a lapse in her insurance coverage, there was no intervention for two years. She was subsequently seen two years after the initial clinic encounter when she returned, insisting on UFE.

**Figure 1:**
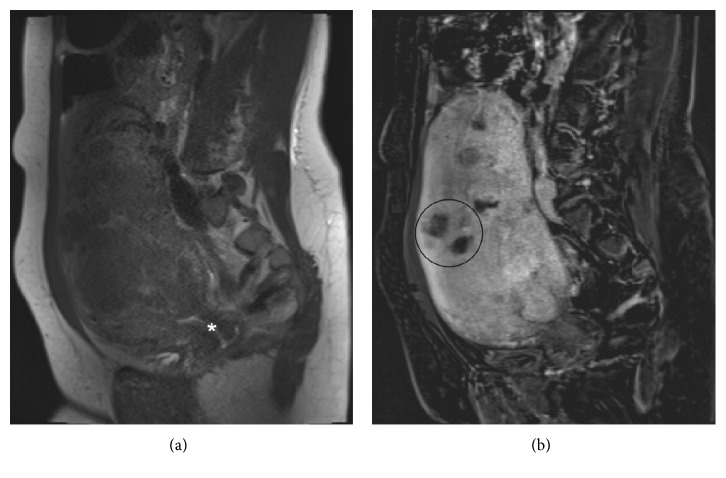
Sagittal view of pre-UFE pelvic MRI (a) prior to and (b) after gadolinium contrast injection. An enlarged uterus, at the level of the cervical canal (asterisk), contains numerous fibroids, with heterogeneous enhancement. One such midanterior mass is annotated (circle).

**Figure 2:**
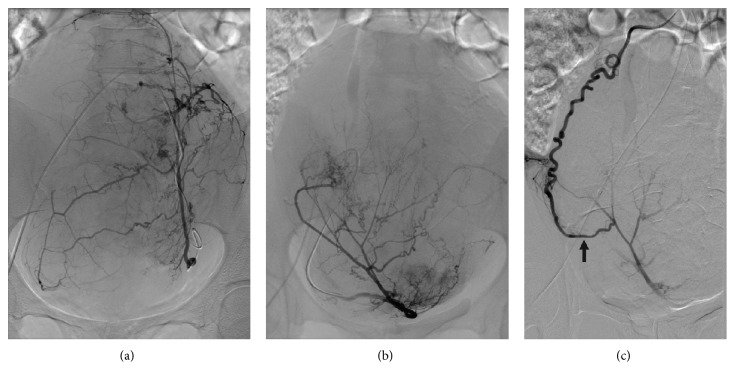
Left (a) and right (b) uterine and (c) right ovarian arteriographic images. (a) Enlarged left uterine artery supplying more than half of the enlarged uterus, with no discrete differentiation of the numerous hypervascular myomas. (b) Enlarged right uterine artery, with similar findings, albeit in a smaller degree, of the left side. (c) Following UAE, tortuous and enlarged right ovarian artery shows continuity with a superolateral branch of the right uterine artery, via a prominent uteroovarian anastomotic arterial branch (black arrow). Not embolized, this ovarian artery maintains some intrauterine flow following fibroid embolization.

Pelvic MRI performed two weeks prior to UFE (Figure 1) depicted approximately twenty fibroids, predominantly intramural in location. The largest mass measured approximately 7.3 × 5.2 × 6.9 cm. There were no fibroids within the distorted endometrial cavity. No extrauterine arterial contribution to the fibroids was reported. Patient underwent UFE (Figure 2) without procedural or immediate postprocedural complications. The left uterine artery was embolized with six vials of 500–700 *μ*m and two vials of 700–900 *μ*m microspheres (Embospheres, Merit Medical Systems, South Jordan, UT, USA). The right uterine artery was embolized with three vials of 500–700 *μ*m microspheres. Both arteries were embolized to near stasis. Completion abdominopelvic aortography showed, with right being greater than left, ovarian arterial contribution to the uterine masses. Stagnation of flow in the opacified intrauterine right ovarian arterial territory obviated the need to reembolize this already embolized territory.

**Figure 3:**
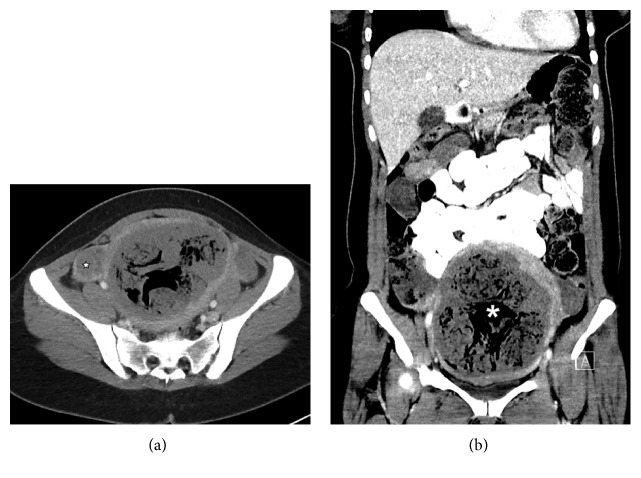
Pelvic CT performed on postprocedure day #26. Axial image (a), at the level of the ovaries (star), and (b) coronal image of the uterus show a large amount of intrauterine air (asterisk), including nonlinear, extravascular air, raising the suspicion of pyomyoma in the proper clinical setting. Patient also had unexpected small bilateral pleural effusions and moderately sized pericardial effusion (not shown).

On postprocedural day (PPD) #10, the patient presented to the emergency department with a 4-day history of fevers up to 103.7°F. The patient also had associated lightheadedness, diaphoresis, and dizziness. She reported a 1-day history of nonradiating right pelvic pain and vaginal spotting with nonmalodorous discharge. In the emergency room, her maximum temperature was 102.7°F. The complete blood count was positive for elevated white blood count of 14.6 K/*μ*L (normal range: 3.7–11.4 K/*μ*L). An absolute neutrophil count was also elevated, 11.5. The hemoglobin was 9.4 g/dL [normal, 12.5–15.5 g/dL]. An abdominopelvic CT showed multiple globular foci of air in multiple necrotic leiomyoma (Figure 3).

Blood and urine cultures were collected. The patient was treated with antibiotic medications, observed for one day, and then discharged. On PPD #13, the patient was recalled and admitted for positive blood cultures, gram-positive cocci in anaerobic bottles. Subsequent blood culture analysis revealed no growth. During the hospital course, she continued to have recurrent fevers on intravenous vancomycin (Hospira, Austin, TX) and zosyn (Pfizer, New York, NY). As seen during the UFE procedure, persistent right hydroureteronephrosis from the enlarged uterus led to a clinical suspicion of associated infection. On HAD #2, she was transfused with two units of packed red blood cells, with Hgb response from 8.8 to 12.4 g/dL. On hospital admission day (HAD) #3, a ureteral stent was placed via cystoscopy, with the patient declining a hysterectomy, despite gynecologic and interventional radiologist's counsel about ongoing fevers, bacteremia, increasing leukocytosis, and the risk of sepsis.

On HAD #4 (PPD #18), the degree of leukocytosis and neutrophilia increased to 24.3 K/*μ*L and 21.4, respectively, despite the intravenous antibiotic regimen. The patient was taken to the operating room for an uncomplicated exploratory laparotomy, supracervical hysterectomy, and bilateral salpingectomy. A 20-week size myomatous uterus with purulent discharge was found. The uterus was bulky and boggy, with normal appearing fallopian tubes and ovaries. Endometrial culture showed moderate white blood cells and rare gram-positive cocci in pairs. The patient was discharged at postoperative day #4. The pathologic specimen revealed endometrium with ulceration, acute and chronic endometritis, and abscess in the myometrium. Embolization material was seen in leiomyomata, with extensive infarction. Fallopian tubes showed acute inflammation, congestion, and edema. Endocervical tissue appeared benign.

Patient had a benign immediate postsurgical convalescence and she was discharged to home on postsurgical day #4. The leukocytosis and neutrophilia normalized over a period of three days, with predischarge values of 7.4 and 7.9, respectively.

## 3. Discussion

Pyomyoma, a suppurative myoma of the uterus, is a rare complication of leiomyoma that has been reported in the postpartum setting and postgynecological procedures and following uterine artery fibroid embolization [1, 6–10]. Most cases of pyomyoma reported in the gynecological literature arise spontaneously, postpartum, or postsurgical. The route of postpartum gynecologic infection is often an ascending infection from the genital tract [8]. After UAE, the route of infection often includes direct spread from the endometrial cavity, spread from adjacent structures such as the adnexae or the bowel, and hematogenous or lymphatic spread [1]. Pyomyoma is often polymicrobial and offending organisms include gram-positive, gram-negative, and anaerobic bacteria [2, 11]. Postembolization syndrome (fever, pelvic pain, and leukocytosis) and vaginal discharge, one week following UFE, without a source of infection, should raise clinical concern for pyomyoma. A triad of sepsis, leiomyoma, and no other source of infection has been described as a concerning clinical presentation for pyomyoma [2].  Table 1 compares this case with the other cases reported in the literature to date [3–7]. Patients' age ranged from 37 to 65 years. Time of the start of the onset of symptoms varied from 6 to 56 days following UFE. There was no identifiable risk factor for post-UFE pyomyoma. The most common presentation in all 5 cases is fever, pelvic pain, and leukocytosis. Reported fevers were higher than that expected with only the postembolization syndrome. Three (60%) of the 5 reported cases presented with leukocytosis, whereas 2 patients presented with a normal white blood cell count. There was no commonality of the reported bacterial organisms. In our study patient, the culture of the surgical specimen was negative, with the findings on imaging and pathologic evaluation suggestive of pyomyoma and pyometra. Our patient had a long exposure to intravenous antibiotics prior to the surgical intervention. In addition to intravenous antibiotics, the majority of the reported patients were treated with hysterectomy, with 1 patient successfully treated with uterine sparing surgical drainage and lavage. 

There was no commonality among the type and amount of embolic materials used for the 5 other reported cases. Only Shukla et al. [5] reported using 500–700-micron microspheres. Neither the type nor the amount of embolic material has been reported as a risk factor for post-UFE infection, a rare and sporadic event [12]. Uterine ischemia has been proposed as a potential contributor to post-UAE infection [5], but, in our patient, this was unexpected as the patient had bilateral ovarian artery contribution to the uterus, neither of which was embolized.

On imaging, visualization of gas in the uterus and uterine vessels is an expected finding and does not necessarily indicate superinfection. This complicates imaging diagnosis of pyomyoma. Air may be seen within the fibroid(s) following UAE. This finding is thought to be the result of gas filling potential spaces left by tissue infarction desiccation. This can be seen as early as one month after UFE and it has been described as a normal finding without signs of infection [11], in contrast to a globular or localized gas collection of a necrotic infected leiomyoma [13]. The specificity of this finding has not however been proven and it remains difficult to diagnose pyomyoma on all current imaging modalities. The most common radiologic finding in the reported cases [3–5, 7] is the presence of an abundance of intrauterine air, with the exception of Pinto et al. who reported a moderate amount of free pelvic fluid [6] on ultrasound, without a corresponding CT examination. This is outlined in Table 2. Quantification and characterization of intrauterine air may be more objective by CT scanning than ultrasound, whereas the latter does not utilize ionizing radiation.

Thus, a strong clinical acumen and a high index of suspicion are imperative. Prompt diagnosis is essential as pyomyoma can lead to serious complications such as myometrial rupture, sepsis, and death. Hysterectomy or myomectomy is the treatment of choice for pyomyoma, although there have been several reports of more conservative surgical drainage [6, 8], including a case of nonsurgical management of clostridial pyomyoma [14].

In summary, this case depicts a rare potentially life-threatening complication, suppurative myoma, and postuterine artery embolization in a young woman. Imaging can be supplementary to diagnose this complication, but diagnosing with imaging alone is difficult given the presence of postprocedural gas up to one month following embolization. Signs and symptoms of postembolization syndrome over one week as well as the triad of fevers, leiomyoma, and no other source for infection should raise clinical concern for pyomyoma.

## Figures and Tables

**Table 1:** 

Author(s) and time of report (year)	Patient age (years)	Prior pelvic infection	Start of symptoms after UAE (PPD #)	Embolic material used	Bacterial organism(s)	Treatment (+ intravenous antibiotics)
Kitamura et al. (2005) [3]	Not reported	Not reported	10	Not reported	Not reported	Not reported

Abulafia et al. (2010) [4]	48	Not reported	7	Not reported	*Escherichia coli *	Hysterectomy

Shukla et al. (2012) [5]	65	Not reported	14	500–700-*μ*m microspheres	*Enterococcus faecalis *	Hysterectomy and bilateral salpingooophorectomy

Pinto et al. (2012) [6]	36	Not reported	56	Not reported	*Propionibacterium acnes *	Laparoscopic drainage and lavage

Rosen et al. (2013) [7]	47	*Trichomonas vaginalis *	6	Not reported	*Staphylococcus aureus*, *Prevotella melaninogenica*	Hysterectomy and bilateral salpingooophorectomy

Current	37	None	6	500–900-*μ*m microspheres	No growth after 1 week	Hysterectomy and bilateral salpingooophorectomy

**Table 2:** 

Author(s) and time of report (year)	Postprocedure time of imaging (weeks)	Sonographic imaging findings	CT imaging findings
Kitamura et al. (2005) [3]	1.5		Increased volume of gas within both infarcted fibroid and surrounding myometrium compared with prior CT scans

Abulafia et al. (2010) [4]	1	Poorly defined mass containing air	15 × 15-cm mass distended with pockets of gas

Shukla et al. (2012) [5]	2		Enlarged mottled-appearing uterus with air in endometrial cavity. No free fluid in pelvis

Pinto et al. (2012) [6]	8	Posterior/fundal myoma measuring 68 × 56 × 55 mm that had become subserosal and a moderate amount of fluid in the pelvis. No adnexal masses	

Rosen et al. (2013) [7]	0.9		Enlarged heterogeneous uterus containing a large amount of myometrial air surrounding multiple enhancing leiomyomas. Myometrial air extended from the lower uterine segment to the fundus

Current	1.5		Multiple globular foci of air in multiple necrotic leiomyoma
